# Tailoring Carbon Quantum Dots via Precursor Engineering for Fluorescence-Based Biosensing of *E. coli*

**DOI:** 10.3390/bios15100635

**Published:** 2025-09-24

**Authors:** Maryam Nazari, Alireza Zinatizadeh, Parviz Mohammadi, Soheila Kashanian, Mandana Amiri, Nona Valipour, Yvonne Joseph, Parvaneh Rahimi

**Affiliations:** 1 Department of Applied Chemistry, Faculty of Chemistry, Razi University, Kermanshah 6714414971, Iran; s.kashanian@razi.ac.ir; 2 Research and Development Division, Danab Knowledge-Based Company, Taq-e-Bostan Boulvard, Kermanshah 6714963488, Iran; arzinati10@gmail.com; 3 Department of Biology, School of Sciences, Razi University, Kermanshah 6714414971, Iran; 4 School of Engineering and Energy, College of Science, Technology, Engineering and Mathematics, Murdoch University, Murdoch 6150, Australia; parviz.mohammadi@murdoch.edu.au; 5 Laboratory for Life Sciences and Technology (LiST), Faculty of Medicine and Dentistry, Danube Private University (DPU), Viktor-Kaplan-Straße 2, Geb. E, EG Nord, 2700 Wiener Neustadt, Austria; mandana.amiri@dp-uni.ac.at; 6 Institute of Nanoscale and Biobased Materials, Faculty of Materials Science and Technology, Technische Universität Bergakademie Freiberg, 09599 Freiberg, Germany; nona.valipour@student.tu-freiberg.de (N.V.); yvonne.joseph@esm.tu-freiberg.de (Y.J.)

**Keywords:** *Escherichia coli*, carbon quantum dot, precursor, fluorescence, detection

## Abstract

Rapid and accurate bacteria identification, particularly *Escherichia coli* (*E. coli*), is essential in the monitoring of health, environment, and food safety. *E. coli*, a prevalent pathogenic bacterium, serves as a key indicator of food and water contamination. Carbon quantum dots (CQDs) have appeared as promising fluorescent probes because of their small size, ease of synthesis, low toxicity, and tunable fluorescence using different carbon-rich precursors. Advances in both bottom-up and top-down synthesis procedures have enabled precise control over CQD properties and surface functionalities, enhancing their capabilities in biosensing. Among the critical factors influencing CQD performance is the strategic selection of precursors, which determines the surface chemistry and recognition potential of the resulting nanodots. The integration with other nanomaterials and the surface modification of CQDs with specific functional groups or recognition elements further improves their sensitivity and selectivity toward *E. coli*. This review summarizes recent progress in the modification of CQDs for the fluorescent detection of *E. coli*, highlighting relevant sensing mechanisms and the influence of different precursors, such as antibiotics and sugars, as well as various functionalization and surface modification strategies. The aim is to provide insight into the rational design of efficient, selective, and cost-effective CQD-based biosensors for bacterial detection.

## 1. Introduction

One of the crucial issues in the field of medical, food, and environmental monitoring is bacteria identification. Traditional detection methods often require specific recognition elements, specialized operators, and lengthy culturing for each bacterial type. In contrast, biosensor technologies, particularly those based on the principle of fluorescence, have emerged as efficient alternatives due to their rapid response, user-friendliness, and high specificity. *E. coli*, with a length of ~1.6 μm and width of ~0.6 μm, is one of the most widespread types found in water and food samples. According to the World Health Organization’s (WHO’s) guidelines, the presence of *E. coli* in water samples with a concentration ranging from 10 to 100 colony-forming units per milliliter (cfu/mL) is indicative of an intermediate risk level of contamination [1]. Also, *E. coli* is one of the major pathogenic microorganisms that can contaminate food products during manufacturing, storage, transporting, and consumption [2].

**Figure 1:**
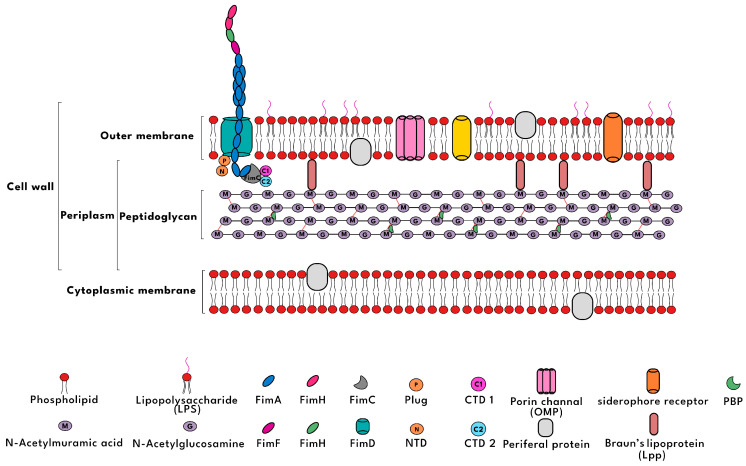
A comprehensive schematic presentation of the cell wall structure.

A critical component of the development of selective biosensors for *E. coli* involves a comprehensive understanding of the structure and physiology of its cell wall. The bacterial cell wall is a dynamic structure that participates in various biological interactions, molecular exchanges, and adaptive responses to environmental changes [3]. These biological functions expose diverse functional groups on the bacterial surface, offering potential binding sites that are particularly relevant for biosensing applications [4]. As demonstrated in Figure 1, *E. coli*, a model Gram-negative bacterium, features a multilayered cell envelope comprising an outer membrane, a thin peptidoglycan layer and periplasmic proteins, and an inner cytoplasmic membrane. This structure is rich in diverse functional groups that serve as key recognition sites for biosensors, including negatively charged phosphate groups from phospholipids and teichoic acids in the peptidoglycan layer, carboxyl groups from lipopolysaccharides (LPS) and peptidoglycan cross-links, as well as amine and hydroxyl groups from outer-membrane proteins (e.g., OmpA) and polysaccharide chains in LPS. These polar and charged moieties enable electrostatic interactions, hydrogen bonding, and specific ligand–receptor-like binding with sensor elements. Notably, LPS in the outer membrane contributes prominently to the bacterium’s negative surface charge and facilitates immunogenic interactions with sensing elements. A comprehensive understanding of these structural attributes is essential for the rational design and surface functionalization of nanomaterials, such as CQDs, with complementary chemical groups. This, in turn, enables their specific and sensitive recognition of *E. coli* in biomedical and food safety applications [5].

CQDs have several unique properties such as a small size, tunable fluorescence, low toxicity, and ease of generation. Therefore, they have been extensively used in research fields including photocatalysis, optoelectronics, bioimaging, and sensing. CQDs can be produced from various carbon-rich organic sources during different synthesis procedures. Also, they exhibit high mechanical strength, conductivity, optical transparencies, narrow bandgaps, high chemical stability, and excellent biocompatibility [6]. Two major synthetic methods, namely “top-down” and “bottom-up”, are used for CQD preparation [7]. The first method involves physical, mechanical, chemical, or electrochemical routes to directly breaking down larger bulky carbon-rich materials (e.g., graphite powder, carbon fibers, graphene sheet, carbon nanotubes, etc.) and synthesizing nanoscale CQDs using successive redox reactions. In contrast, the second method is based on the pyrolysis or carbonization of small-molecule soluble precursors (e.g., citric acid, sucrose, glucose, aromatic molecular structures, benzene, etc.). Oxidation methods, arc discharge, laser ablation, and ultrasonic-assisted methods are used as top-down procedures, and the pyrolytic process, template-assisted route, microwave-assisted synthesis, and solvothermal/hydrothermal carbonization are four main subclasses of bottom-up approaches. Because of the use of nontoxic precursors, cost reduction, and more accurate controlling of reaction parameters, bottom-up procedures have attracted great attention in recent years for CQD synthesis [8].

The fluorescent, chemical, and physical properties, including fluorescence lifetime, emission wavelength, quantum yield, responsiveness to specific substances, and biocompatibility, are governed by the presented functional groups on CQDs’ surfaces [9]. The targeting capability of CQDs can be tailored during synthesis by incorporating recognition-active precursors such as antibiotics, sugars, or heteroatom dopants. These modifications strengthen electrostatic or covalent interactions with bacterial cells, thereby improving the sensitivity and selectivity of the resulting fluorescence sensors. Meanwhile, the employment of antibodies, aptamers, and other excellent specific recognition elements in the post-synthesizing modification of CQDs demonstrates remarkable efficacy in the construction of high-performance fluorescent sensing systems [10].

In this review, we summarize the latest advances in the application of CQDs for fluorescence sensing *E. coli.* This review focuses on how specific precursors employed during CQD synthesis can promote selective or strong bacterial binding, and also focuses on post-synthetic modifications with biorecognition elements that can enhance detection specificity. In addition, the underlying interaction mechanisms between CQDs and *E. coli* are discussed. This review aims to provide valuable insights into the rational design of high-performance CQD-based sensors, encouraging further research into targeted *E. coli* detection through the selection of tailored precursors and surface engineering.

## 2. Fluorescence *E. coli* Sensing Assay Using CQDs

CQDs, with superior photostability, narrow emission spectra, and high quantum yields compared to traditional fluorophores, enable sensitive *E. coli* detection through multiple fluorescence-sensing mechanisms, including aggregation-induced emission (turn-on), aggregation-caused quenching (turn-off), inner filter effect (IFT), Förster resonance energy transfer (FRET), and photo-induced electron transfer (PET) [10,11,12,13].

The sensing ability of CQDs is strongly influenced by their abundant surface functional groups, such as carboxyl (–COOH), carbonyl (–CO), hydroxyl (–OH), and amino (–NH_2_), which facilitate electrostatic and hydrogen-bonding interactions with cellular components [14]. As already noted, in *E. coli*, the negatively charged LPS of the outer membrane and polar moieties of membrane proteins act as major binding sites. Binding events modulate the local electronic environment of CQDs, resulting in fluorescence quenching (turn-off), enhancement (turn-on), or spectral shifts. Moreover, given that CQDs are highly sensitive to environmental parameters such as pH, polarity, and viscosity, their photoluminescence serves as a responsive probe for characterizing bacterial presence and activity [15,16]. A representative example was reported by Kumari et al., who synthesized CQDs from single-use plastics for selective turn-off sensing of *E. coli* [16]. Increasing bacterial concentrations led to a significant decrease in fluorescence intensity due to interactions between CQD surface groups and outer-membrane proteins of *E. coli*. The binding process also affected the radiative recombination rate of photo-generated electrons and holes within the CQDs, further amplifying the fluorescence quenching signal and enabling bacterial detection.

Despite these advantages, CQD-based sensing still faces challenges such as limited selectivity and potential interference from non-target species, which can trigger similar quenching effects. To overcome these limitations, researchers have explored a variety of strategies, including heteroatom doping to enhance quantum yield and introduce selective binding sites, integration with other nanomaterials to amplify fluorescence responses, and precursor engineering with sugars or antibiotics to tailor surface chemistry for bacterial affinity. Moreover, the functionalization of CQDs with biorecognition elements such as antibodies and aptamers has proven to be particularly effective for achieving highly specific interactions with *E. coli* [10]. Such modifications have resulted in the development of hybrid fluorescent probes with significantly enhanced sensing performance, allowing for the faster, more selective, and more sensitive detection of bacterial targets such as *E. coli*.

## 3. Hybrid CQDs Material by Integrating Other Nanomaterials

**Figure 2:**
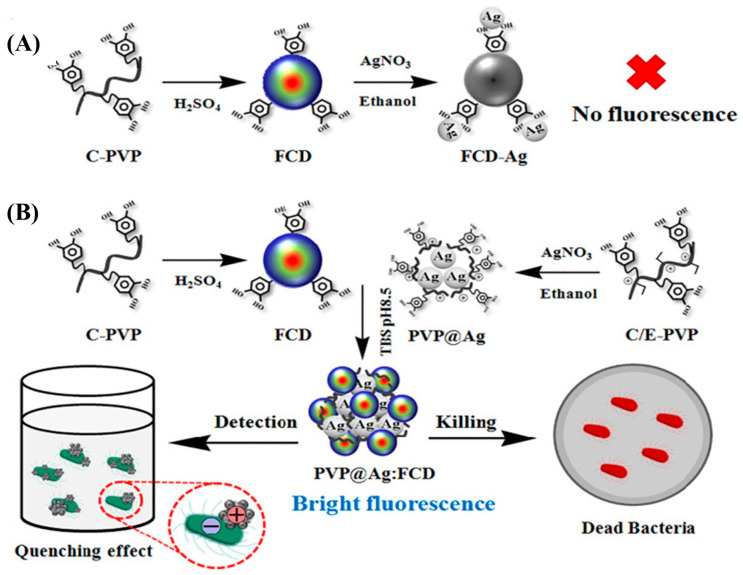
(**A**) Illustration of Ag-induced fluorescence quenching; (**B**) illustration of synthesis of PVP@Ag/CQD (in original paper shown as PVP@Ag:FCD) via catechol cross-linking for bacterial detection and killing [20]; reprinted with permission from Elsevier.

Integrating CQDs with other nanomaterials, particularly metal nanoparticles, is an effective strategy for enhancing the optical properties, stability, and sensing performance of *E. coli* detection [17,18,19,20,21]. Bhaisare et al. [17] reported a hybrid system consisting of magnetic nanoparticles (Fe_3_O_4_) as the core, decorated with CQDs, forming Fe_3_O_4_-CQDs. Characterization of the core-shell Fe_3_O_4_-CQDs confirmed that the CQDs were uniformly coated onto the Fe_3_O_4_ without altering their crystal phase. This stability is attributed to van der Waals forces and electrostatic interactions between the two components. The Fe_3_O_4_-CQDs were further functionalized with chitosan to introduce amine groups for bacterial sensing. The resulting amine-functionalized Fe_3_O_4_-CQDs demonstrated a high level of sensitivity and a strong affinity for detecting of *E. coli* and *S. aureus*. In addition to acting as effective fluorescent probes, their magnetic properties enabled the efficient separation and collection of bacterial cells. Furthermore, the surface amine groups enabled selective binding through electrostatic attraction to negatively charged bacterial cell surfaces. The limit of detection (LOD) for *E. coli* and *S. aureus* was found to be 3.5 × 10^2^ cfu/mL and 3 × 10^2^, respectively, consistent with MALDI-MS analysis results. In another study, core–shell Fe_3_O_4_-CQDs were synthesized via a hydrothermal method using turmeric, lemon, and grapefruit extracts, along with ethylenediamine as precursors and Fe_3_O_4_ as the magnetic core [19]. These hybrid nanomaterials were then used as a rapid and sensitive platform for quantitatively detecting *E. coli*, showing a fluorescent response over a wider concentration range (0 to 9 × 10^7^ cfu/mL) compared to a previous report [17]. Recently, a dual-functional material combining CQDs and silver nanoparticles (AgNPs) has been developed for the sensitive detection, as well as the eradication, of *E. coli* and *S. aureus* [20] (Figure 2). Although AgNPs are widely recognized for their potent antibacterial properties, their association with CQDs can result in fluorescence quenching through FRET, which could affect sensing performance. To address this issue, a polyvinylpyrrolidone (PVP) polymer coating was applied to the AgNPs (PVP@AgNPs) using catechol chemistry. CQDs were synthesized by carbonizing a catechol-functionalized quaternized PVP polymer, and subsequently conjugating it with PVP@AgNPs to yield catechol-crosslinked PVP@Ag/CQD composites. In the presence of bacteria, electrostatic interactions occurred between the positively charged regions of the PVP@Ag/CQDs and the negatively charged LPS on the bacterial surface. This was followed by the hydrophobic interaction-driven aggregation of the bacterial membrane and the hydrophobic domains of the composite, resulting in aggregation-induced fluorescence quenching. The extent of the quenching effect depended on the concentration of bacteria present, enabling the quantification of *E. coli* and *S. aureus* over a wide detection range 1–10^7^ cfu/mL. Alongside their role as fluorescent sensors, the AgNPs within the composifigurete contributed to a strong antibacterial effect. Upon contact, the AgNPs accumulated at the bacterial membrane, disrupting its integrity through aggregation and ultimately inducing bacterial cell death, even at low concentrations.

Hybridizing CQDs with other nanomaterials, particularly magnetic nanoparticles and metal nanoparticles (e.g., Ag, Au), significantly enhances the fluorescence mechanisms, as well as the biosensing performance, of *E. coli* detection by leveraging synergistic properties like magnetic separation and plasmonic amplification. Nevertheless, significant challenges remain in developing green and scalable synthesis methods and in designing real-time, multiplexed detection platforms for diverse bacterial strains. Integrating machine learning to optimize nanomaterial–CQD interactions and design multi-color CQD arrays offers a promising direction to overcome these hurdles, ultimately enabling cost effective and sustainable biosensors for applications in food safety and environmental monitoring.

## 4. Precursor-Driven Functionalization CQDs for Selective *E. coli* Recognition

As mentioned above, a significant factor in determining the specificity and sensitivity of CQDs in biosensing applications, particularly for the detection of *E. coli*, is the nature of the precursors used in synthesis. These precursors directly influence the surface chemistry, functional groups, and optical behavior of CQDs, thereby shaping their interaction with bacterial targets. Series of molecules, such as urea and citric acid, etc., are generally utilized as foundational carbon sources to fabricate the CQDs for sensing of the most analytes including *E. coli* [15].

However, CQDs derived from these fundamental precursors frequently exhibit constrained selectivity, attributable to the deficiency of functional groups that would enable specific bacterial recognition. To address this limitation, heteroatom doping, particularly with nitrogen and sulfur, has become a widespread method to enhance both the fluorescence properties and the binding affinity of CQDs toward *E. coli*. Furthermore, molecules with inherent bacterial recognition capabilities, such as antibiotics (widely used as antimicrobial agents) and sugars (key nutrients for bacteria that bind to lectins on the bacterial surface), can serve not only as functionalizing agents, but also as precursors in the one-step synthesis of CQDs. When used as precursors, these molecules can dope the CQDs without the need for additional doping agents, and they can also retain partial structural motifs on CQD surfaces. This enables the resulting CQDs to retain specific recognition properties towards bacterial targets, offering a promising approach to targeted bacterial detection. These approaches are discussed in more detail in the following sections. Overall, the selection and engineering of CQD precursors can be regarded as a powerful strategy for developing high-performance *E. coli* fluorescent sensors.

### 4.1. Heteroatom Atom Doping (N, B, S) in CQD Structures

The doping of CQDs with heteroatoms such as nitrogen (N), phosphorus (P), boron (B), and sulfur (S) has emerged as an effective strategy to tune their physicochemical and optical properties. The incorporation of these atoms into the carbon framework not only alters the fluorescence characteristics, but also introduces functional groups that enhance the CQDs’ interaction with target biomolecules like *E. coli*. Pathak et al. [22] synthesized N-doping CQDs (N-CQDs) using citric acid and glycine using the hydrothermal method. The N-CQDs exhibited strong pH-sensitive fluorescence, with maximum intensity at pH 2, attributed to the protonation of pyridinic and pyrrolic nitrogen, which enhances local emission states. At this acidic pH, *E. coli* cells typically possess a zeta potential of −18.4 mV, while the protonated N-CQDs demonstrated a less negative potential of −8.4 mV. This reduced electrostatic repulsion facilitated closer interactions between the N-CQDs and *E. coli*, enhancing the fluorescence response. In another study, L. Zhang et al. [4] synthesized fluorescent N-/B-co-doped CQDs (N,B-CQDs) using L-tartaric acid, L-arginine, and boric acid. Arginine functioned as the nitrogen source, contributing positively charged guanidino groups that promoted electrostatic interactions with the negatively charged surface of *E. coli*. Simultaneously, boronic acid groups formed reversible covalent bonds with cis-diol groups present in the saccharide residues of LPS located on the bacterial outer membrane. This dual-binding mechanism, electrostatic adsorption combined with boronate ester formation, facilitated the rapid and stable attachment of the nanoprobes to bacterial cells [23]. As a result, the N-/B- co-doped CQDs enabled sensitive *E. coli* detection, yielding a linear concentration range of 10^2^–10^7^ cfu/mL and a LOD of 165 cfu/mL.

### 4.2. Using Antibiotics for CQDs Fabrication/Functionalization

In order to design targeted CQD-based fluorescence sensors for the detection of *E. coli* using antibiotics, a fundamental understanding of the mechanisms of interaction between antibiotics and bacteria is required. Antibiotics are key agents in treating bacterial infections and function by penetrating bacterial cell envelopes and disrupting vital physiological processes. For instance, β-lactam antibiotics enter the periplasmic space via outer-membrane porins, where they inhibit peptidoglycan transpeptidase enzymes. This effectively blocks cell wall synthesis, leading to bacterial death. In contrast, polymyxin antibiotics such as colistin bind specifically to LPS in the outer membrane of Gram-negative bacteria. This interaction destabilizes the membrane, enabling the antibiotic to penetrate the inner membrane and disrupt its integrity, ultimately resulting in cell lysis. By conjugating such antibiotics to CQDs, these fluorescence nanoprobes gain an enhanced affinity for bacterial surfaces through electrostatic and molecular recognition interactions. In addition to surface functionalization, numerous efforts have been made to utilize antibiotics as precursors for the one-step synthesis of CQDs, offering a more convenient approach for practical applications. Due to their chemical composition, which contains not only carbon but also elements such as N, S, and oxygen (O), antibiotics can serve as both a carbon source and a doping agent, eliminating the need for additional precursors. Furthermore, portions of their molecular structure may be retained in the final CQDs, thereby preserving their ability to recognize bacteria. These targeted strategies enable more specific and sensitive fluorescence responses, facilitating the rapid detection of bacteria like *E. coli* in complex biological or environmental samples [24,25,26].

Table 1 summarizes the primary mechanisms by which antibiotics enter bacterial cells and interact with specific cellular targets, as well as their relative efficacy against Gram-negative bacteria [27,28,29,30,31]. Gaining this insight is valuable for guiding the design of targeted sensing agents and improving diagnostic strategies.

In this regard, several studies have attempted to develop antibiotic-modified CQDs to achieve the more specific and sensitive optical detection of *E. coli*. Amikacin, a biologically active synthetic aminoglycoside antibiotic, and colistin, a multicomponent polypeptide antibiotic, are among the most frequently employed ligands for the functionalization of CQDs, significantly enhancing their specificity and selectivity toward *E. coli* detection [32,33,34,35,36,37,38,39]. Chandra et al. [32] were the first to report the selective detection of *E. coli* using fluorescent CQDs conjugated with amikacin. The amikacin-modified CQDs were synthesized via hydrothermal carbonization using amikacin and diammonium hydrogen citrate as precursors, with varying amikacin concentrations. Fourier Transform Infrared (FTIR) analysis confirmed the presence of –COOH functional groups and amide bonds on the surface of the amikacin-modified CQDs, indicating the successful integration of the antibiotic into the carbon matrix. Elemental analysis and energy-dispersive X-ray (EDX) spectra showed that the modified CQDs had a higher nitrogen content than the unmodified ones, suggesting effective N-doping originating from amikacin during hydrothermal synthesis. Furthermore, photoluminescence measurements revealed a red shift in the excitation wavelength of the modified CQDs, which also supports the incorporation of N-containing moieties from the antibiotic. The CQDs synthesized with higher amikacin content demonstrated efficient and selective detection of *E. coli* over a wide linear concentration range, achieving a LOD of 552 cfu/mL. This enhanced performance can be attributed to the increased presence of amide and –COOH groups, along with N-doping, which collectively enhance photoluminescence and enable robust electrostatic and hydrogen-bonding interactions with *E. coli*’s negatively charged LPS and polar outer-membrane proteins, resulting in a more robust fluorescence response. Remarkably, the sensor maintained high selectivity, even in the presence of *S. aureus* and in complex matrices such as fruit samples. Due to limitations in the scalability of the synthesis process, the authors developed an alternative approach involving the conjugation of CQDs with colistin, an antibiotic known to interact specifically with LPS. This interaction displaces divalent cations from the phosphate groups of membrane lipids, destabilizing the LPS membrane and ultimately causing bacterial cell death. To achieve this, a simple pilot-scale solid-state carbonization method was employed, using ammonium citrate as a carbon source and varying amounts of colistin sulfate to produce colistin-functionalized CQDs. [33]. FTIR analysis confirmed the presence of –COOH and –OH functional groups, as well as amide bonds forming on the CQD surface. This indicated that the colistin has successfully been conjugated. X-ray Photoelectron Spectroscopy (XPS) analysis revealed that CQDs synthesized with a higher colistin content exhibited increased nitrogen and oxygen levels, which correlated with enhanced fluorescence intensity. Furthermore, ^1^H-NMR and zeta potential measurements provided additional evidence of successful surface modification with colistin. The resulting fluorescent probes, particularly those synthesized with higher colistin concentrations, demonstrated the highly sensitive detection of *E. coli* with a lower LOD of 460 cfu/mL in complex real samples such as human urine, apple juice, and tap water. This increased efficiency arose from the synergistic effects of colistin’s strong affinity for LPS, the improved surface functionality of the CQDs, and enhanced fluorescence properties resulting from nitrogen and oxygen doping. These factors collectively facilitate stronger binding interactions and more sensitive fluorescent responses towards *E. coli*.

Putri et al. [34] developed a highly sensitive fluorescence sensor for *E. coli* detection using a dual strategy. This approach combined the co-doping of CQDs with N and S atoms (N,S-CQDs) and then functionalizing them with the antibiotic amikacin. The amikacin-conjugated N,S-CQDs were synthesized via a microwave-assisted method using citric acid as the carbon source, thiourea as the nitrogen and sulfur dopant, and varying amounts of amikacin as the targeting moiety. Doping with N and S, which provide extra electrons for n-type doping, promoted radiative relaxation pathways and created new excitation energy traps. This enhanced the fluorescence intensity. Additionally, incorporating amikacin into the N,S-CQDs significantly improved their affinity and selectivity towards *E. coli*. FTIR and ^1^H-NMR data confirmed the successful conjugation of amikacin to the N,S-CQD surface via amide bond formation between the –OH groups of the CQDs and the –NH_2_ groups of amikacin. The fluorescence emission spectra of the amikacin-modified N,S-CQDs revealed multiple emissive traps arising from various surface states with different energy levels. These were attributed to the presence of various O-, N-, and C-containing functional groups on the surface. Detection of *E. coli* was achieved through electrostatic interactions between the positively charged amino groups of amikacin and the negatively charged LPS on the bacterial surface. This interaction enabled the sensor to achieve an exceptionally low LOD of 1.5 cfu/mL.

**Figure 3:**
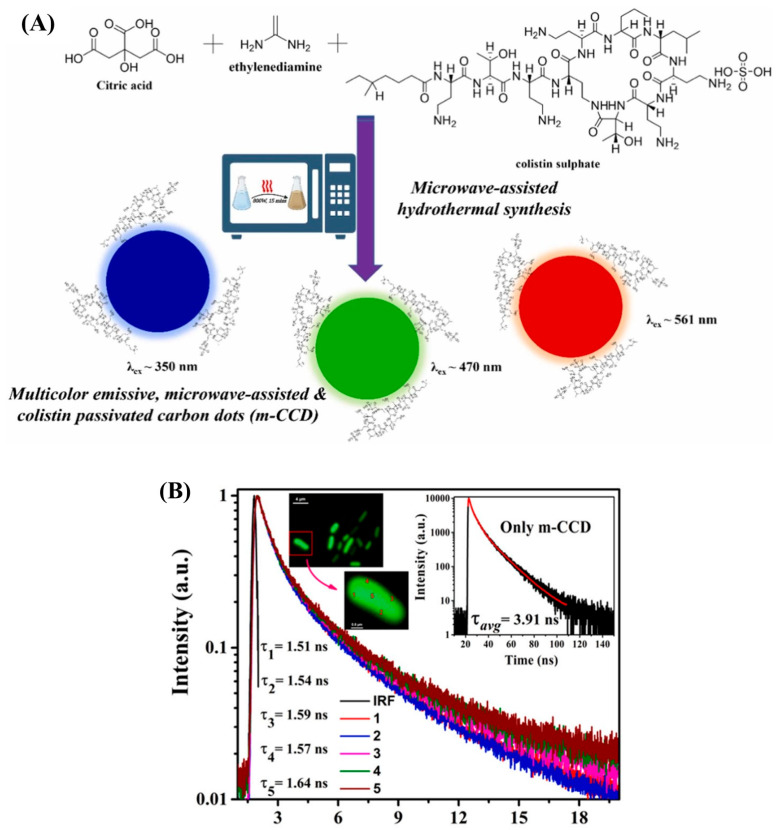
(**A**) Illustration of the microwave-assisted hydrothermal synthesis of m-CCQD having surface passivated colistin moieties with multi-emissive properties; (**B**) curves of FLIM-based point lifetime decay of individual m-CCD-treated *E. coli* measured on the cell wall (1, 2, 3, and 4) and cytoplasm (5) (inset: lifetime decay of free m-CCD in solution) [35]; reprinted with permission from Elsevier.

Recently, an assay for *E. coli* detection was developed using multi-emissive colistin-passivated CQDs (m-CCQDs), based on fluorescence lifetime (FLT) response and Fluorescence Lifetime Imaging Microscopy (FLIM) [35]. This approach enabled not only the sensitive detection of *E. coli*, but also provided insights into the interaction between the fluorescence probe and bacterial cells. The m-CCQDs were synthesized via a one-step microwave-assisted hydrothermal process using citric acid, ethylenediamine (as an N-doping agent), and colistin (Figure 3A). The time-resolved fluorescence properties of m-CCQDs vary depending on their local environment due to their multi-emissive nature, ranging from picoseconds to hundreds of nanoseconds. FLIM was used to measure changes in FLT between free m-CCQDs and m-CCQD-treated *E. coli*, with a specific focus on the outer-membrane and cytoplasmic regions (Figure 3B). Effective binding was indicated by fluorescence emission when m-CCQDs interacted with bacterial cells. This binding is attributed to the polycationic nature of the colistin moiety, which enables it to interact selectively with Gram-negative bacteria such as *E. coli*. Colistin disrupts the outer membrane by displacing divalent cations (e.g., Mg^2+^ and Ca^2+^), which stabilize LPS. This leads to pore formation and increased membrane permeability. This enables the colistin-passivated CQDs to penetrate the bacterial cell. A notable reduction in fluorescence lifetime was observed upon binding, decreasing from 3.91 ns for free m-CCQDs to 1.57 ns in the presence of *E. coli*. This substantial decrease is likely due to the increased non-radiative processes resulting from the interactions between the m-CCQDs and the bacterial components. The assay enabled quantification of *E. coli* across two linear ranges: 3.40 × 10^5^–9.80 × 10^5^ and 6.90 × 10^7^–4.14 × 10^8^ cfu/mL. Its performance was reliable in both standard bacterial cultures and real samples, including tap water and human urine.

In most reported studies, CQDs are often functionalized with antibiotics for bacterial detection. However, Kuang et al. employed a different approach, synthesizing CQDs using a rapid, one-step, microwave-assisted method involving varying reaction powers and using cefminox sodium (CS), a potent antibiotic, as the single precursor [36]. UV–vis absorption spectroscopy revealed that, during synthesis, the carboxyl groups of CS participated in intermolecular reactions that contributed to stabilizing the hydrothiazine-conjugated structure. Furthermore, the S atom attached to the tetrazole ring may have facilitated intermolecular cross-linking under microwave heating due to its lone pair electrons. XPS analysis confirmed this, revealing an S–S bond absorption peak likely originating from cross-linking among S atoms on the CS side chains. Thanks to the mild reaction conditions, several functional groups from the original CS molecule were retained in the CS-CQDs, including the hydrothiazine and tetrazole rings. This structural preservation endowed the CQDs with desirable characteristics, including excellent water solubility, antibacterial activity, and low biological toxicity. The CS-CQDs formed a dual-function platform with diagnostic and therapeutic capabilities. They successfully detected *E. coli* in blood samples in a short timeframe, overcoming the limitations of conventional diagnostic methods which typically require several days. In both in vitro and in vivo models, E. coli was quantitatively detected within approximately six hours across a concentration range of 0.5 × 10^6^–1 × 10^9^ cfu/mL.

### 4.3. Using Sugars for CQD Fabrication/Functionalization

As sugars are a primary nutrient source for bacteria and exhibit a natural affinity for bacterial surface lectins through interactions, modifying CQDs with sugars is a promising strategy for the selective detection of pathogens such as *E. coli* [40]. Specifically, the FimH lectin subunits located on the type 1 pili of wild-type *E. coli* demonstrate a particularly high binding affinity for mannose (Man) compared to other sugars, such as glucose or lactose. FimH binds to Man using its Man-binding domain, which interacts through hydrogen bonds and van der Waals forces [41]. Consequently, most reported sugar-conjugated CQD probes for *E. coli* detection have utilized mannose as the functional precursor in the synthesis of sugar-modified CQDs.

**Figure 4:**
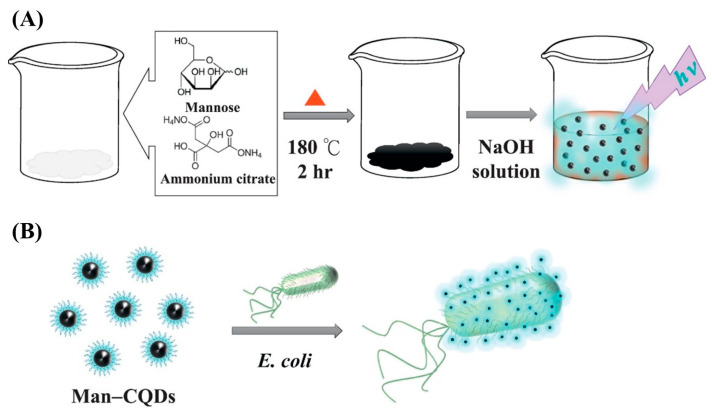
(**A**) The synthesis of Man-modified CQD-based fluorescent probes; (**B**) their use in the selective detection of *E. coli* [42]; reprinted with permission from Elsevier.

Weng et al. [42] were the first to synthesize and biofunctionalize a CQD-based fluorescent probe by directly pyrolyzing of ammonium citrate in the presence of Man (Figure 4). XPS measurements confirmed the presence of O and N atoms in the Man-N,CQDs. The Man-N,CQDs demonstrated excitation-dependent fluorescence, with maximum emission wavelengths at 360 nm and 440 nm. This excitation dependency is indicative of the presence of CQDs with different hybridized compositions and structures, varying emissive trap sizes, and a distribution of surface states. The resulting Man-N,CQDs were then employed for the selective detection of *E. coli*. To evaluate the specificity of the Man-N,CQDs, a series of sugar-conjugated N,CQDs were synthesized using the same method, namely, by using glucose-, fructose-, sucrose-, and lactose-conjugated N,CQDs. As expected, these alternative sugar-modified N,CQDs exhibited significantly lower fluorescence responses towards *E. coli*, which is attributed to their weaker binding affinity to the bacterial FimH lectin. In contrast, the Man-N,CQDs demonstrated strong and selective binding, highlighting the superior affinity of Man for FimH and confirming its effectiveness as a targeting ligand in *E. coli* sensing with LOD of 450 cfu/mL. However, a key limitation of this study was the unclear nature of the interaction between Man and the CQDs, specifically whether Man was physically adsorbed onto the CQD surface or covalently bound through dehydration reactions during the carbonization of ammonium citrate. To address this ambiguity, a two-step synthesis approach was developed. In this method, the CQD was synthesized independently first, followed by biofunctionalization with Man through a dehydration coupling reaction between the –COOH groups on the CQD surface and the–OH groups of the Man [43]. This strategy enabled the formation of a well-defined core–shell structure, with the Man acting as a functional shell around the CQD core. Importantly, this method also enabled precise control over the density of Man on the CQD surface, thereby improving the consistency and efficiency of bacterial targeting. Compared to previous study [42], the proposed probe enabled highly sensitive quantification of *E. coli*, achieving a lower LOD of 100 cfu/mL.

In another study, instead of directly using Man as a biofunctionalization agent, papaya peel, a natural carbon source rich in Man, was utilized to synthesize water-soluble and ethanol-soluble CQDs (W-CQDs and E-CQDs, respectively) [44]. The FTIR spectra of raw papaya powder, W-CQDs, and E-CQDs revealed the presence of N–H, –COO, C–O, and C–H functional groups on their surfaces. This finding was subsequently confirmed using XPS analysis. The analysis of the particle size distribution indicated that the mean particle size of W-CQDs and E-CQDs was approximately 3.4 nm and 10.8 nm, respectively. The reduced size of W-CQDs is likely attributable to the presence of saccharides and other water-soluble micromolecules, which promote the formation of more compact CQDs. Conversely, the larger size of E-CQDs was attributed to the presence of more organic macromolecules in ethanol, which resulted in the formation of larger particles. Functionally, the W-CQDs exhibited a significant fluorescence enhancement across a wide concentration range of *E. coli* (10^5^–10^8^ cfu/mL), with an LOD of 9.5 × 10^4^ cfu/mL. Conversely, the E-CQDs exhibited no discernible fluorescent response to *E. coli*. This disparity is ascribed to the saccharide composition, specifically Man, in the W-CQDs, which are capable of interacting with FimH lectins present on the surface of *E. coli* within aqueous environments. Meanwhile, the E-CQDs, predominantly comprising organic macromolecules (a consequence of their larger size), exhibited a lack of such interactions, and, consequently, did not display a comparable fluorescent response.

Although Man-modified CQDs offer a selective fluorescence probe for *E. coli* sensing, their specificity can be compromised in complex biological matrices or when multiple bacterial species are present, as Man-binding lectins may also be expressed by other microorganisms, potentially leading to cross-reactivity. To improve the poor specificity in this case, multi-functionalization is needed, involving the integration of additional targeting ligands. However, this approach introduces new challenges, including increased synthesis complexity, potential interference between functional groups, and the requirement to preserve the optical and biocompatible properties of the CQDs. Alternatively, bacterial sugar metabolism offers a selective sensing pathway, as it not only causes changes in the pH of the culture medium, but also varies among different bacterial species [10]. In this context, Yang et al. [45] synthesized pH-sensitive fluorescent CQDs via the carbonization of sucrose with sulfuric acid. The presence of –OH and –C=O functional groups on CQD surfaces was confirmed through FTIR analysis. Transmission electron microscopy (TEM) images revealed that the CQDs were spherical in shape. To evaluate the use of the CQDs as a ratiometric pH probe for bacterial detection, *E. coli* cells were cultured in a lactose-selective medium, and the corresponding pH changes at different cell densities were monitored using a pH meter. Subsequent to the incorporation of the pH-sensitive fluorescent CQDs into the bacterial culture, the fluorescence intensities at two emission wavelengths (410 nm and 350 nm) were measured. The fluorescence intensity ratio exhibited a proportional increase in accordance with the concentration of *E. coli*, thereby demonstrating a linear detection range from 13 to 1.33 × 10^5^ cfu/mL and an extremely low LOD of 1 cfu/mL. This assay was successfully used to detect *E. coli* in milk and sewage water, and the results were validated using MALDI-TOF MS.

As mentioned earlier, different bacteria exhibit distinct sugar metabolism pathways, resulting in varying pH changes. This insight can be leveraged to design a system capable of identifying and distinguishing between bacterial species simultaneously. Another study, Zhao et al. developed pH-sensitive fluorescent CQDs via a one-step hydrothermal synthesis using o-phenylenediamine and dopamine [46]. The fluorescence intensity of these CQDs varied with pH: under basic conditions, the surface amino groups (–NH_2_) remained unprotonated, resulting in strong fluorescence. However, under acidic conditions, protonation led to significant fluorescence quenching. Since bacteria such as *E. coli* and *S. aureus* can metabolize sugars like glucose and lactose to produce acidic byproducts, adding CQDs to cultures containing these bacteria decreased the fluorescence intensity. Specifically, the CQD-based system achieved LOD of 21 cfu/mL for *E. coli* and 33 cfu/mL for *S. aureus* in the presence of glucose. To enable simultaneous detection, they exploited the selective metabolism of lactose, which is only decomposed into acid by *E. coli*, by designing two systems. One system contained CQDs, glucose, and a mixture of *E. coli* and *S. aureus*, while the other contained CQDs, lactose, and the same bacterial mixture. This strategy allowed for differential fluorescence responses based on the species of bacteria present, allowing for dual identification in mixed samples.

Table 2 summarizes a range of CQD-based fluorescence sensors, their synthesis methods, and the corresponding linear ranges and LODs for *E. coli* detection. From these studies, it can be noted that precursor-driven functionalization is among the most versatile strategies for tailoring CQDs toward selective *E. coli* recognition. Heteroatom doping (e.g., N, B, S) can effectively tune surface states and enhance binding affinity, although challenges remain in terms of reproducibility and precise control over doping levels. Antibiotic-derived CQDs, such as those synthesized from amikacin, demonstrate strong specificity, but raise concerns regarding resistance development and long-term biocompatibility. In contrast, sugar-based CQDs from natural precursors (e.g., glucose, citrus extracts) represent a promising green and low-cost alternative, although their stability and selectivity under complex real-sample conditions still require further optimization.

## 5. Post-Synthetic Biofunctionalization of CQDs for Target Specificity

**Figure 5:**
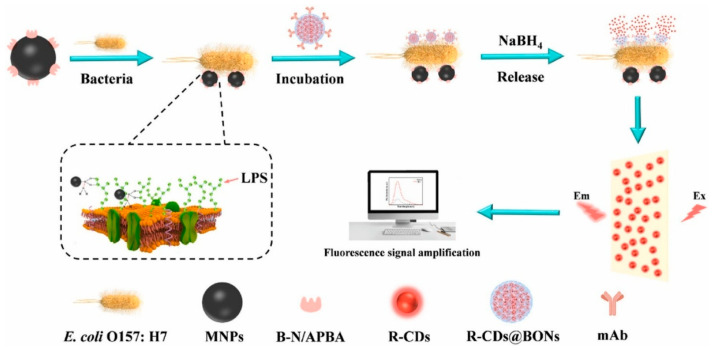
Fluorescence signal amplification biosensor for ultrasensitive detection of *E. coli* [49]; reprinted with permission from Elsevier.

In addition to making CQDs more specific through the design of selective precursors, the functionalization of CQDs with high-affinity biorecognition elements offers a promising strategy for developing highly selective fluorescence-based bioassays for bacterial detection. Consequently, considerable efforts have recently been directed towards modifying CQDs, particularly with natural antibodies and their artificial counterparts, aptamers, in order to improve the selectivity and sensitivity of bacterial detection assays. The functionalization of CQDs with antibodies provides an exceptionally specific and sensitive immunoassay of recognizing bacteria, owing to the inherent high affinity and selectivity of antibodies towards specific bacterial antigens. Zhao et al. [47] introduced a cell-based fluorescent microsphere system using inactivated Staphylococcus aureus cells as carriers and CQDs as fluorescent reporters for the selective detection of *E. coli* in milk. The specificity of the microspheres was attributed to Staphylococcal Protein A (SPA) present on the surface of *S. aureus* cells, which exhibits a high affinity for antibodies and enables their directional immobilization. In this approach, anti-*E. coli* antibodies were conjugated to the microspheres, which were subsequently combined with immunomagnetic separation for fluorescence-based detection of sandwich-structured immunocomplexes. The developed assay enabled selective quantification of *E. coli* over a concentration range of 2.4 × 10^2^–2.4 × 10^7^ cfu/mL within 30 min. The direct immobilization of antibodies on the surface of CQDs poses significant challenges due to their nanoscopic dimensions compared to larger nanoparticles. To overcome this limitation, Song et al. [48] developed CQD-encapsulated silica nanospheres. The silica matrix served a dual purpose: it enhanced the structural stability and fluorescence intensity of the CQDs, and it provided a more favorable surface for effective antibody immobilization. This design enabled the construction of a highly selective sensing platform for quantifying *E. coli*. The sensor demonstrated a wide linear detection range of 0–10^4^ cfu/mL and an ultra-low LOD of 2.4 cfu/mL. Recently, a sandwich-type magnetic separation-based fluorescence signal-amplification biosensor was developed for the ultrasensitive detection of *E. coli*, utilizing two distinct nanosystems [49] (Figure 5). The first system comprised red fluorescent CQDs encapsulated within breakable organosilica nanocapsules (BONs), which were conjugated to anti-*E. coli* monoclonal antibodies, forming Ab@CQDs@BONs. The second system comprised Wulff-type boronic acid-functionalized magnetic nanoparticles (MNPs@B–N/APBA), which exhibited broad-spectrum bacterial recognition capabilities and enabled the specific capture of *E. coli*. These two systems together formed a sandwich complex (MNPs@B–N/APBA∼*E. coli*∼Ab@CQDs@BONs), facilitating the quantitative detection of *E. coli* over a concentration range of 10^1^–10^6^ cfu/mL. The assay demonstrated a low LOD of 25 cfu/mL in both pure cultures and artificially contaminated lettuce samples, with a total detection time of approximately 120 min. Very recently, Wang et al. [50] proposed a simplified and rapid immunofluorescence assay for the specific detection of *E. coli*, utilizing a FRET mechanism. In this design, CQDs functioned as fluorescence donors, while covalent organic frameworks (COFs) served as fluorescence acceptors. The CQDs were bioconjugated to *E. coli*-specific antibodies (CQDs-Ab), which then interacted with the COFs. Upon interaction, the COFs underwent a morphological transformation from spherical to irregular structures with multiple protrusions, while the fluorescence lifetimes of the CQDs remained largely unchanged. This indicated a static quenching effect, supporting the occurrence of FRET between the CQDs-Ab and the COFs. The specificity of the system for *E. coli* was demonstrated by a marked increase in fluorescence intensity within 30 min, in contrast to other bacterial strains. A linear relationship was found between the concentration of *E. coli* (ranging from 0 to 10^6^ cfu/mL) and the restored fluorescence intensity of the CQDs. The LOD was remarkably low at 7 cfu/mL. This restoration was attributed to disruption of the CQDs-Ab–COF complex upon specific binding with *E. coli*, leading to the progressive recovery of the CQD fluorescence signal.

Although immunoassays offer enhanced specificity, their broader application is hindered by several limitations, particularly in the detection of pathogens within complex and contaminated matrices [51]. These include the loss of antibody-binding affinity at elevated temperatures, thermal-induced aggregation, low thermal stability, and poor solubility. Aptamers, which are synthetic mimics of antibodies composed of 10–50 nucleotides of single-stranded DNA (ss-DNA) or RNA, are a promising alternative. They demonstrate comparable or even superior efficiency, while offering greater stability, reproducibility, and adaptability under harsh conditions. In line with this, a fluorescence-based bioassay was developed using CQDs functionalized with aptamers and MNPs labeled with complementary DNA (cDNA-MNPs) [52]. The system operated via a fluorescence quenching mechanism in the presence of *E. coli*: the fluorescence intensity of the CQDs is progressively reduced upon target binding. The assay enabled the quantitative detection of *E. coli* within the range of 500 to 10^6^ cfu/mL, achieving a detection limit of 487 cfu/mL. In another study, a FRET-based sensing platform was developed for *E. coli* detection, employing aptamer-functionalized CQDs as the fluorophore and graphene oxide (GO) as the quencher [53]. GO was chosen due to its excellent quenching efficiency and high repeatability in FRET systems. Fluorescence quenching occurred via hydrophobic interactions and π–π stacking between the aptamer-modified CQDs and the GO surface. When *E. coli* was introduced, specific binding between the aptamer and the target bacteria caused the CQDs to detach from the GO surface, significantly restoring fluorescence intensity. This assay demonstrated a detection limit of 89 cells/mL and exhibited a linear response within the range of 10^2^–10^7^ cells/mL. Recently, Bai et al. developed two *E. coli* detection assays based on FRET [54] and the IFE [55] mechanisms. In the FRET-based assay, CQDs functionalized with *E. coli*-specific aptamers served as fluorescence donors, while AgNPs acted as acceptors [54]. The fluorescence of the aptamer-bound CQDs was quenched in the presence of AgNPs due to FRET. Upon the introduction of *E. coli*, the formation of the aptamer–*E. coli* complex led to the dissociation of CQDs from the AgNPs, resulting in the recovery of fluorescence. This assay enabled the specific detection of *E. coli* across a wide linear range of 2 × 10^3^–2 × 10^8^ cfu/mL, with a low LOD of 77 cfu/mL. In the second strategy, Bai et al. [55] employed the IFE as the detection mechanism. Unlike FRET, the IFE does not require direct chemical or covalent interactions between the fluorophore and the absorber. Instead, it operates based purely on spectral overlap, specifically the absorber’s ability to reduce the excitation or emission light of the fluorophore when their spectra overlap. This makes IFE-based assays comparatively simpler and more cost-effective to implement. In this approach, an aptamer specific to *E. coli* was adsorbed onto the surface of AgNPs, stabilizing them in a dispersed state. Upon the introduction of CQDs, the dispersed AgNPs effectively quenched the fluorescence of CQDs via the IFE mechanism due to the spectral overlap between the absorption wavelengths of the AgNPs and the excitation/emission wavelengths of the CQDs. However, in the presence of *E. coli*, the aptamers bind preferentially to the bacterial cells, thereby reducing their availability to stabilize the AgNPs. Under high-salt conditions, this results in the aggregation of the AgNPs, altering their optical properties and diminishing their absorption in the relevant spectral region. Consequently, the aggregated AgNPs are no longer able to efficiently quench the fluorescence of the CQDs, resulting in a fluorescence recovery that correlates with the concentration of *E. coli*. This IFE-based sensor demonstrated a wide linear detection range of 2 × 10^2^ to 2 × 10^7^ cfu/mL, with a detection limit as low as 185 cfu/mL.

Some fluorescence sensors for *E. coil* sensing based on CQDs that are functionalized by an antibody and aptamer are summarized in Table 3. Post-synthetic biofunctionalization with aptamers and antibodies represents a transformative approach for CQD-based biosensors, leveraging molecular precision to achieve exceptional specificity in real-world samples. The ability to target distinct *E. coli* surface components (e.g., LPS, OmpA) enhances diagnostic reliability; however, challenges such as conjugation efficiency and biomolecule stability remain. Looking ahead, progress should focus on cost-effective and stable conjugation strategies, as well as the integration of multi-target aptamer arrays with machine learning. Such developments could enable the multiplexed detection of *E. coli* strains, including antibiotic-resistant variants, thereby supporting robust applications in public health and environmental monitoring.

## 6. Conclusions

This study highlights the status of CQDs as a highly promising class of nanomaterial for the rapid and selective fluorescent detection of *E. coli*, which is a critical indicator of contamination in food and water samples. Their unique physicochemical properties, including tunable fluorescence, low toxicity, and easy functionalization, make them ideal for biosensing applications. A thorough understanding of *E. coli* structure, specially cell wall architecture, is essential for the rational design of selective sensing platforms. The interaction of CQDs with *E. coli* is primarily driven by electrostatic, hydrogen-bonding, and molecular interactions with specific components of the bacterial cell wall, including negatively charged lipopolysaccharides (LPS) and polar outer-membrane proteins, which can be exploited for selective recognition. Advances in precursor selection and doping strategies allow for the precise modulation of surface chemistry, enhancing both sensitivity and specificity. Furthermore, functionalization with complementary nanomaterials or biorecognition elements such as antibodies and aptamers provides additional pathways to improve detection performance and selectivity. Together, these innovations contribute to the development of efficient, cost-effective, and portable CQD-based biosensors with significant potential for use in public health surveillance and environmental monitoring.

Despite these advancements, challenges remain, including scalable green synthesis, consistent performance in complex biological matrices, and the differentiation of antibiotic-resistant *E. coli* strains. Future opportunities lie in integrating machine learning for pattern-based pathogen discrimination, developing multiplexed CQD arrays for multi-strain detection, and advancing ecofriendly precursors to ensure sustainable, cost-effective biosensors for public health and environmental monitoring.

## Figures and Tables

**Table 1:** The primary mechanisms of bacterial entry, interaction with cellular targets, and the relative efficacy of different antibiotic classes against Gram-negative bacteria.

Antibiotic Class	Examples	Mechanism of Entry	Target and Mode of Action	Effectiveness Against Gram-Negative Bacteria
β-lactams and Monobactams	Penicillin, Cephalosporins, Aztreonam	Enter through porins (OmpF, OmpC) or passively diffuse	Target peptidoglycan transpeptidase (PBP) to inhibit cell wall synthesis	Effective, depends on porin access
Polymyxins	Colistin, Polymyxin B	Bind to LPS, alter membrane permeability, penetrate	Disrupt inner membrane integrity, causing cell lysis	Highly effective
Glycopeptides	Vancomycin, Teicoplanin	Cannot penetrate outer membrane due to size	Bind to D-alanine–D-alanine in peptidoglycan, blocking cell wall extension	Ineffective
Lipopeptides	Daptomycin	Unable to pass through outer membrane due to LPS	Targets bacterial membrane but cannot bind to Gram-negative membranes	Ineffective
Fosfomycin	Fosfomycin	Enters via GlpT/UhpT transporters	Inhibits MurA enzyme, first step in peptidoglycan synthesis	Variable, resistance rising in some Gram-negative
Cycloserine	D-cycloserine	Diffuses passively or via porin channels	Inhibits alanine racemase and D-Ala-D-Ala ligase in cell wall synthesis	Limited; used mainly for MDR-TB, weak Gram-neg coverage
Chloramphenicol (Amphenicols)	Chloramphenicol	Passive diffusion (due to small size)	Inhibits protein synthesis by binding to 50S ribosomal subunit	Moderate (some Gram-neg coverage, but resistance concerns)
Tetracyclines	Doxycycline, Tetracycline	Enters via passive diffusion or OmpF/OmpC porins	Inhibits protein synthesis by binding 30S ribosomal subunit	Effective against many Gram-negatives
Aminoglycoside	Amikacin, Gentamicin	Requires O_2_-dependent active transport (porins aid entry)	Binds irreversibly to 30S ribosomal subunit, causing misreading of mRNA	Highly effective against many aerobic Gram-negatives

**Table 2:** A list of CQDs based fluorescence sensors for *E. coil* detection.

CQD Type	Precursor	Synthesis Method	QY %	Linear Range (cfu/mL)	LOD (cfu/mL)	Refs
CQD	Urea, Citric acid, NaOH	Hydrothermal (160 °C, 6 h)	-	-	-	[15]
CQD	Plastic polybags, cups and bottles	Thermal calcination (300 °C, 2 h) followed by hydrothermal (200 °C, 5 h)	60, 65, 69	0–40 × 10^8^	down to 10^8^	[16]
Mag-CQDs	Acetic acid (4%), chitosan, Fe_3_O_4_ NPs	Hydrothermal (180 °C, 12 h)	-	4.0 × 10^2^–3.4 × 10^3^	3.5 × 10^2^	[17]
N,Zn-CQD	Glucosamine, Zinc acetate, water	Hydrothermal (130 °C, 1 h)	74	0.25–125 µM	0.15 µM	[18]
Fe_3_O_4_-CQDs	Magnetite, H_2_O, lemon turmeric	Hydrothermal (180 °C, 6 h)	-	-	-	[19]
CQD	Pvp, CCDP, ethanol, H_2_SO_4_, N_2_	Hydrothermal (70 °C, 10 h)	-	-	-	[20]
CQD	Egg White	Thermal (200 °C, 4 h)	43	0.997–0.999	40 nm (40 ng/mL)	[21]
N-CQDs	Citric acid, glycine	Incubation at 70 °C for 12 h, then Hydrothermal (230 °C, 6 h)	27.2	-	-	[22]
N,B-CQDs	TA, Arg, and H_3_BO_3_	Hydrothermal (180 °C, 10 h)	14.5	10^2^–10^7^	165	[4]
CQDs@amikacin	Di ammonium hydrogen citrate and amikacin	Hydrothermal (180 °C, 4 h)	12.35	7.625 × 10^2^–3.904 × 10^5^	552	[32]
CQDs@ colistin	Di ammonium hydrogen citrate and colistin sulfate	Pyrolysis (180 °C, 1 h)	7.56	3.81 × 10^2^–2.44 × 10^4^	460	[33]
N,S-CQDs@amikacin	Citric acid, thiourea, and amikacin	Microwave-assisted treatment (180 °C, 30 min)	-	0–1.20 × 10^4^	3.04	[34]
CQDs@ colistin	Citric acid, ethylenediamine and colistin sulfate	Microwave-assisted (15 min)	25.1	3.40 × 10^5^–9.80 × 10^5^ and 6.90 × 10^7^–4.14 × 10^8^	3.68–4.89 × 10^4^	[35]
cefminox sodium-CQDs	cefminox sodium	microwave		0.5 × 10^6^–1 × 10^9^	3.7 × 10^5^	[36]
CQDs@ colistin	Ammonium citric and colistin sulfate	Pyrolysis (180 °C, 1 h)	-	10^2^–10^4^	-	[37]
CQD@van	Citric acid, Urea, Water, phosphate buffer, EDC, NHS, Vancomycin	Hydrothermal (Microwave, 750 W)	-	3.18 × 10^5^–1.59 ×10^8^	9.4	[38]
(PM-BA-Van)CQD’s	Ammonium citrate/3-Aminophenylboronic acid, vancomycin hydrochloride/Polymaxin B sulfate	Thermal (4 h, 180)	-	-	-	[39]
Man-CQDs	Ammonium citrate and Man	Pyrolysis (180 °C, 2 h)	9.8	10^2^–10^8^	450	[42]
CQD Man-FCQD FA-CQD	Ammonium citrate for CQD synthesizing; Man and FA for functionalizing	Pyrolysis (180 °C for 2 h) for CQD fabrication; Pyrolysis (180 °C for 2 h) for CQD functionalization with Man and FA	9	0–10^8^ 0–10^8^ 0–10^8^	- 100 -	[43]
W-CQDs	Papaya peel	Hydrothermal (200 °C, 5 h)	18.98	10^5^–10^8^	9.5 × 10^4^	[44]
p-CQD	Sucrose, H_2_SO_4_, NaOH	Hydrothermal	-	-	3.5 × 10^2^	[45]
CQD	Dopamine and oPD	Hydrothermal (200 °C, 8 h)	-	10^3^–10^7^	21	[46]

**Table 3:** A list of fluorescence sensors for *E. coil* detection based on CQDs functionalized by an antibody and aptamer.

CQD Type	Precursor	Synthesis Method	QY %	Linear Range (cfu/mL)	LOD (cfu/mL)	Refs
Ab-CQDs-microsphere	Citric acid, urea, and CaCl_2_	Hydrothermal (250 °C)	-	2.4 × 10^2^–2.4 × 10^7^	2.4 × 10^2^	[47]
Ab–CSN	Aminosalicylic acid	Solvothermal (200 °C, 18 h)	16.4	0–10^4^	2.4	[48]
mAb@R-CDs@BONs-NH2	reduced glutathione, formamide	Hydrothermal (160 °C, 1 h)		10^1^–10^6^	25	[49]
CQD-Ab-COF	Ascorbic acid	Hydrothermal (180 °C, 8 h)	50.8	0–10^6^	7	[24]
NH_2_-CQD-apt + GO	Polyethyleneimine and citric acid monohydrate	Hydrothermal (180 °C for 2 h)	-	10^2^–10^7^	89	[53]
Apt-CQDs + AgNPs	White grapefruit peels and ethylene glycol	Microwave oven (3 min)	-	2 × 10^3^–2 × 10^8^	77	[54]
Apt + CQDs + AgNPs	Celery leaves	Hydrothermal (200 °C, 12 h)	-	2 × 10^2^–2 × 10^7^	185	[55]

## Data Availability

Data sharing is not applicable (only appropriate if no new data is generated or the article describes entirely theoretical research).

## Abbreviations

The following abbreviations are used in this manuscript:

Ab	Antibody
AgNPs	Silver nanoparticles
Arg	L-arginine
BONs	Encapsulated breakable organosilica nanocapsules
cfu/mL	Colony-forming units per milliliter
CQDs	Carbon quantum dots
CQDs-Ab	*E. coli*-specific antibodies
CS	Cefminox sodium
CSNs	CQD-hybridized silica nanospheres
*E. coli*	*Escherichia coli*
EDX	energy-dispersive X-ray
FRET	Förster resonance energy transfer
FTIR	Fourier Transform Infrared
GO	graphene oxide
IFE	Inner filter effect
LOD	Limit of detection
LPS	Lipopolysaccharides
mAb@R-CDs@BONs-NH_2_	R-CDs@BONs labeled with anti-*E. coli* O157:H7 monoclonal antibody
Mag	Magnetic
Man	Mannose
m-CCQDs	multi-emissive colistin-passivated CQDs
MNPs@B–N/APBA	Wulff-type boronic acid-functionalized magnetic nanoparticles
N-CQDs	N-doping CQDs
N,B-CQDs	N-/B-co-doped CQDs
Omp	Outer-membrane protein
oPD	o-Phenylenediamine
PET	Photo-induced electron transfer
PVP	Polyvinylpyrrolidone
SPA	Spectra Staphylococcal Protein A
ss-DNA	single-stranded DNA
TA	L-tartaric acid
W-CDs	Water soluble carbon dots
WHO	World Health Organization
XPS	X-ray Photoelectron Spectroscopy
